# A Two-Dimensional Flow Sensor with Integrated Micro Thermal Sensing Elements and a Back Propagation Neural Network

**DOI:** 10.3390/s140100564

**Published:** 2013-12-31

**Authors:** Ruiyi Que, Rong Zhu

**Affiliations:** State Key Laboratory of Precision Measurement Technology and Instruments, Department of Precision Instruments, Tsinghua University, Beijing 100084, China; E-Mail: qry09@mails.tsinghua.edu.cn

**Keywords:** two-dimensional flow measurement, thermal flow sensor, BP neural network

## Abstract

This paper demonstrates a novel flow sensor with two-dimensional 360° direction sensitivity achieved with a simple structure and a novel data fusion algorithm. Four sensing elements with roundabout wires distributed in four quadrants of a circle compose the sensor probe, and work in constant temperature difference (CTD) mode as both Joule heaters and temperature detectors. The magnitude and direction of a fluid flow are measured by detecting flow-induced temperature differences among the four elements. The probe is made of Ti/Au thin-film with a diameter of 2 mm, and is fabricated using micromachining techniques. When a flow goes through the sensor, the flow-induced temperature differences are detected by the sensing elements that also serve as the heaters of the sensor. By measuring the temperature differences among the four sensing elements symmetrically distributed in the sensing area, a full 360° direction sensitivity can be obtained. By using a BP neural network to model the relationship between the readouts of the four sensor elements and flow parameters and execute data fusion, the magnitude and direction of the flow can be deduced. Validity of the sensor design was proven through both simulations and experiments. Wind tunnel experimental results show that the measurement accuracy of the airflow speed reaches 0.72 m/s in the range of 3 m/s–30 m/s and the measurement accuracy of flow direction angle reaches 1.9° in the range of 360°.

## 1. Introduction

Two-dimensional flow measurement is becoming more and more important in many applications such as meteorology, drag reduction research for aircraft and vessels [1–4], biomedical flow detection [5] and control enhancement for Unmanned Air Vehicles (UAVs)/Micro Air Vehicles (MAVs) [6–9]. Conventional techniques are mostly based on Pitot tubes (including hemispherical nose probes) [10–13] or electromechanical self-orienting vanes [12–14], which usually protrude outside the testing body and disturb the flow they measure, need hard mechanical ties and/or intrusive pneumatic links inside. Their fabrication and packaging processes are generally elaborate and do not meet practical requirements. When applied to small objects, more problems occur, such as big size and heavy weight, high power consumption, difficulty to install, tendency to break and so on. In recent years, many researchers have applied micromachining processes to realize microsensors and arrays for measuring flow vectors [15–24], however, most sensors are still complex, fragile and power consuming.

Ozaki's work [14] showed the measurement of the flow-induced force in 1-Degree Of Freedom (DOF) and 2-DOF sensory hairs. The measuring probe of the 2-DOF sensor was made up of a long wire attached to the center of a cross-shaped beam with strain gauges on the four roots. It could measure the direction angle of air flow with one sensor hair. However, the sensor was hard to fabricate and the structure was fragile and easily broken.

Chen's work [24] contains two types. One is based on an orthodox micromachined Hot Wire Anemometer (HWA). Using a plastic deformation magnetic assembly method, an out-of-plane HWA with two support beams was made. They then combined three orthogonal hot wires together to form a three-dimensional sensor. The other method is based on momentum transfer principles and inspired by fish lateral line sensors, adopting a similar principle as Ozaki's work. The fabrication for realizing these sensors is relatively complex.

The works of Dong *et al.* [25], Kim *et al.* [17], Van Oudheusden *et al.* [23], Furjes *et al.* [21], de Bree *et al.* [22] and Van Oudheusden [19] all used thermal sensing methods to detect two-dimensional flow. They used several heaters and temperature detectors distributed around the center of the chip to detect flow-induced temperature differences. The sensor usually consisted of multiple isolated heaters and thermometers with separate electrical connection pads and thus made the structures relatively complicated and large.

**Figure 1.**
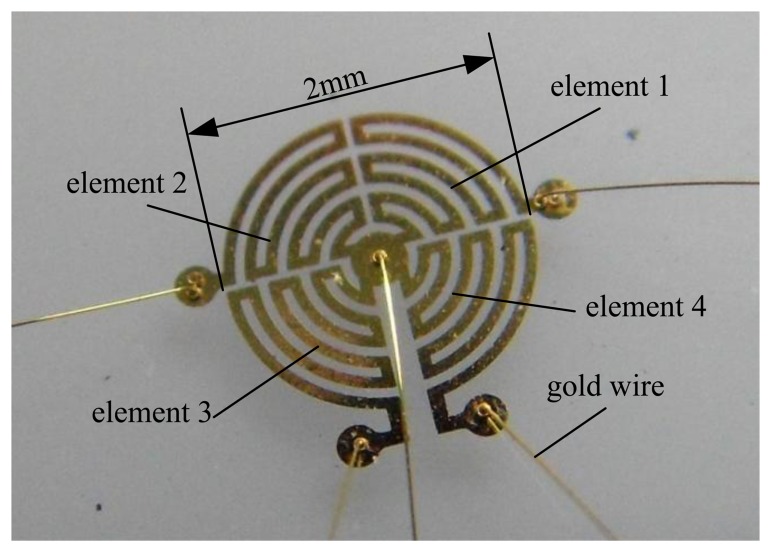
Prototype of hot-film flow vector sensor.

This paper presents a novel flow sensor with a relatively simple structure. As shown in Figure 1, the probe of the sensor is made up of four elements; each of them is composed of a roundabout wire distributed in a quadrant of a circle that serves as both Joule heater and temperature sensor. Each element works in a constant temperature difference (CTD) mode. The readouts of the four sensing elements are used to deduce the flow parameters of the 2-D flow (*i.e.*, flow speed and direction angle) using a neural network data fusion technique. Compared with previous technologies, the sensor has merits of simple structure, low cost, easy fabrication and low power consumption.

## 2. Operation Principle and Design of the Sensor System

The sensor uses thermal elements serving as both Joule heater and temperature sensor so that it has a relative simple structure and low-cost fabrication.

### 2.1. Sensing Principles

**Figure 2.**
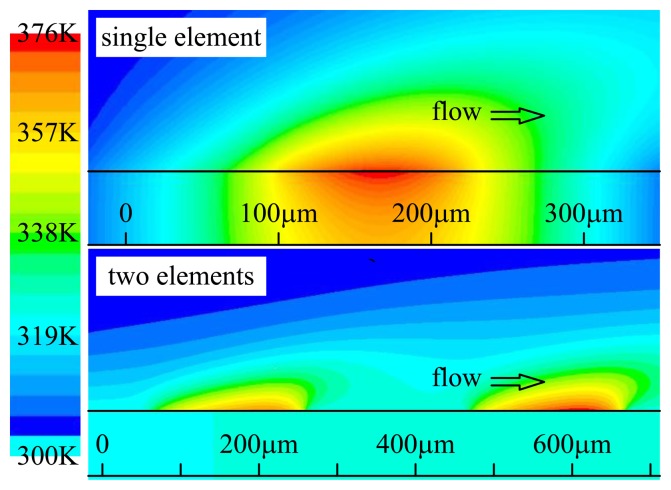
Temperature distribution above the surface of thermal elements.

The working principle of the sensor is based on the heat transfer of the heating element in a flow field [11], which forms a temperature distribution above the thermal element. Under a constant bias power and zero flow speed, the thermal element achieves a steady-state temperature, which means the heat transfer system reaches equilibrium. When an external flow passes through the sensor, the temperature field will be deflected in the direction of the flow that results in the temperature differences among the elements according to their locations of upstream or downstream as shown in Figure 2. Temperature differences among the four elements can be detected and used to figure out the magnitude and direction of the flow.

### 2.2. Sensing Design and Simulation

**Figure 3.**
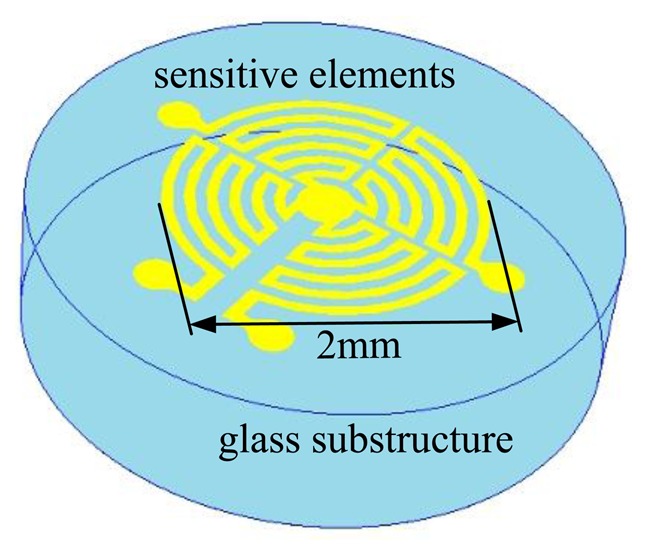
Sensor design.

**Figure 4.**
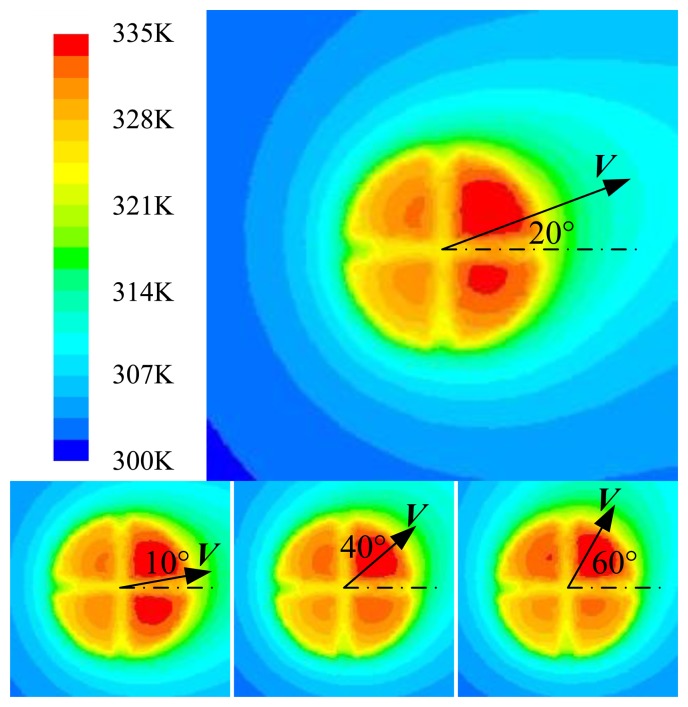
Results of simulation under a flow with different flow directions.

For sensing the 2-D flow in the directional range of 360°, both heating and sensing structures need to follow some requirements. Firstly, the heating structure needs to have central symmetry so as to form a centrosymmetric temperature distribution above the sensor, specifically a circular symmetry is an optimal option for covering 360° in all directions. Secondly, the temperature sensing structure needs to be divided into several isolated sections to detect the flow-induced temperature differences. For integrating the heating and sensing elements into one element, we consider the use of a round shape and divide it equally into several sections. The number of divided sections gives the number of heating/sensing elements, which also determines the number of conditioning circuits needed to operate the heating and temperature sensing. For simplifying the operation and saving energy, the number of heating/sensing elements needs to be minimized. After overall considerations, we divide the round shape into four equal sections as shown in Figure 4, each of which is a quadrant consisting of a roundabout wire, as shown in Figures 1 and 3.

The sensitive area of the sensor needs to be as small as possible so as to capably detect the local flow at one point. The diameter of the sensitivity element is designed to be 2 mm and the roundabout element wire has a width of 75 μm and a gap distance of 75 μm considering about the feasibility of fabrication.

Heating up the four quadrants by applying an equal constant electrical power to each quadrant, a circular symmetric temperature distribution is formed. When a flow passes through the sensor, the temperature field will be deflected in the flow direction and generates the temperature differences among the four quadrants. The simulated results using *ANSYS FLUENT* under a flow with different direction angles are shown in Figure 4.

### 2.3. Sensitive Element and Fabrication

**Figure 5.**
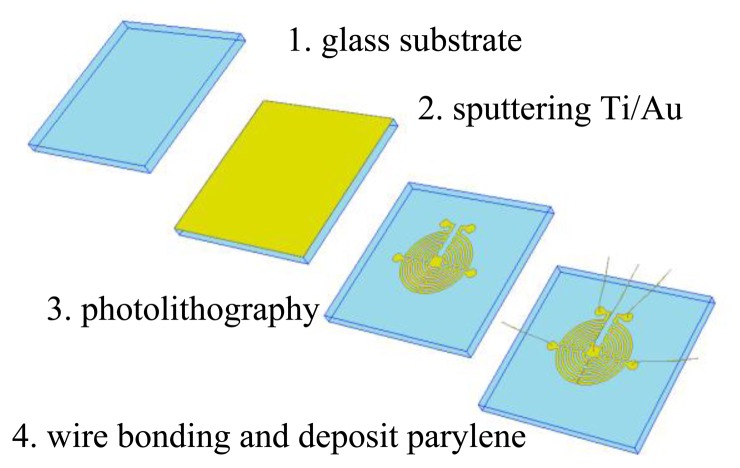
Diagram of the fabrication process of the sensor prototype.

The elements of the sensor are fabricated using a simple lift-off micromachining process shown in Figure 5. A 400μm thick polished glass wafer is used as substrate. The process starts with sputtering Ti/Au film (100 nm), which is then patterned to form the wire elements using photolithography. Gold is selected as the material of the sensor elements because it has good thermoelectricity and conductivity for realizing the integration of the sensor elements, electric wires and pads. Afterwards the four element wires are electrically connected to the external electrical circuit via wire bonding; herein only five pads are needed for the sensor (the central pad is a shared ground of the four elements). Finally, a parylene film (10 nm) is deposited on the wafer and served as an encapsulation.

The temperature coefficients of resistance (TCR) of the fabricated sensing elements are tested to be about 2,000 ppm/K, and the resistances of the elements are around 35 Ω.

### 2.4. Conditioning Circuit

**Figure 6.**
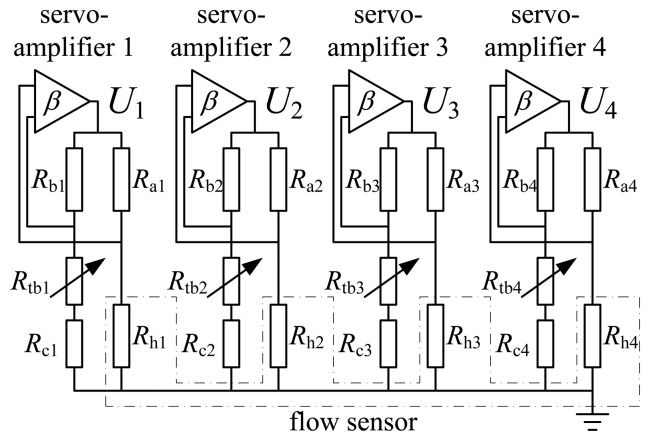
Scheme of the conditioning circuits.

The sensor is operated in constant temperature difference (CTD) modes with a built-in temperature compensation. The CTD mode takes merits of the high sensitivity and fast dynamic response. The temperature compensation is realized by putting a temperature sensor (e.g., Pt100) into the resistor bridge circuit of the anemometer and adopting a balance design to figure out the resistors of the bridge for implementing temperature compensation [26]. In CTD mode, a feedback is employed to maintain a constant temperature difference between the element and ambient fluid for the thermal flow sensor. Scheme of CTD mode conditioning circuits for operating the flow vector sensor is shown in Figure 6. It consists of four CTD units sharing a common ground (the central pad shown in Figure 1). In Figure 6, *R*h1–4 are the four elements of the sensor. *R*c1˜4 are the temperature resistors used for temperature compensation, *R*tb1–4 are the resistors used to adjust Joule heating of *R*h1–4, *R*a1–4 and *R*b1–4 are the resistors used for balancing the bridges. The resistor bridge voltages *U*1, *U*2, *U*3, *U*4 (*i.e.*, heating voltages) are the outputs of the sensors. For realizing the temperature compensation, the resistors in the bridge need to follow the relationship [26]:

(1) $$\frac{R_{\text{a}}}{R_{b}}=\frac{R_{c0}\cdot \alpha_{c}}{R_{h0}\cdot \alpha_{h}}$$

(2) $$R_{tb}=R_{c0}\left(\frac{\alpha_{c}}{\alpha_{h}}+\alpha_{c}\cdot \Delta T-1\right)$$

where *R*h0 denotes the resistance of the flow sensor *R*h at 0 °C, *αh* denotes the TCR of *R*h, *ΔT* denotes the temperature difference between the sensor element and the ambient fluid, *Rc*0 denotes the resistance of the temperature sensor *R*c at 0 °C, *α_c_* denotes the TCR of the temperature sensor *R*c. *R*a and *R*b are the resistors used for balancing the bridges.

Using the above operation circuit, the time constant of the sensing element is tested to be less than 15 ms and the sensitivity of the element is estimated to be around 0.4 V^2^/(m/s)^0.5^ at the overheat ratio of 10% according to King's law [11]. The tested speed resolution reaches 0.1 m/s, and the power consumption of each element is tested to be about 40 mW for an overheating ratio of 10%.

## 3. BP Neural Network-Based Data Fusion for 2-D Flow Measurement

### 3.1. Basic Principles

Intuitively, the variation of the average temperature of the sensor elements depends on the flow speed, while the temperature differences among the four elements rely on the flow direction. However, the facts are complex, as changing either of the flow speed or direction results in variations of the average temperature and temperature differences among the thermal elements. In addition, the individual differences among the fabricated four elements will also induce the complication of the relationship between the readouts of the sensor and flow parameters. An effective data fusion technique needs to be studied to decouple the problem and figure out the flow parameters (speed and direction denoted as [*V*, *θ*]). Under CTD mode, the temperatures of the sensing elements remain constant by modulating the heating power (*i.e.*, the heating voltages of the four elements denoted as [*U_1_*, *U_2_*, *U_3_*, *U_4_*]), which are output readings of the sensors.

### 3.2. Configuration of the BP Neural Network System

The relationship between the sensors' readouts and the flow parameters is a multiple input multiple output (MIMO) coupling system, which can be formulated by:

(3) $$\left[V,\theta ]=f(U_{1},U_{2},U_{3},U_{4}\right)$$

**Figure 7.**
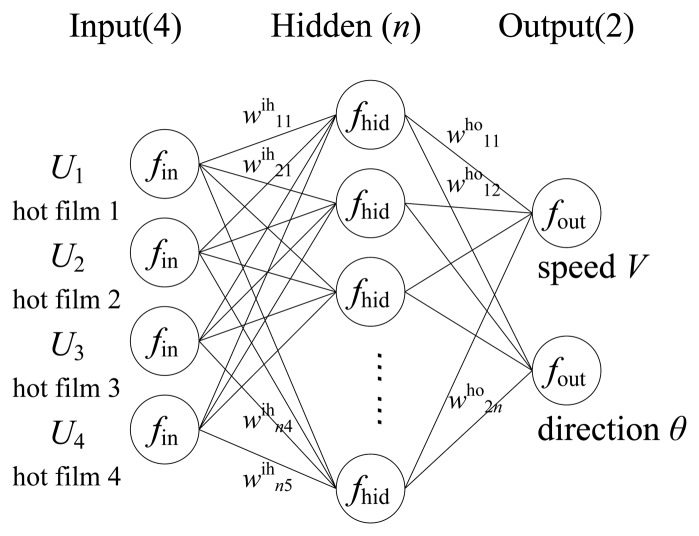
Structure of the neural network.

As matter of fact, the function *f* is generally a nonlinear function depending on the structure and material of the sensor. In this paper, we adopted an experiment-based model identification method to develop the model *f* and used a 3-layer Back Propagation (BP) neural network as the model structure. It had been theoretically proved that three layers of neural network could solve arbitrarily complicated nonlinear mapping problems [27]. The structure of the network is shown in Figure 7.

The number of hidden neurons is determined through experimental testing (in our work we determined the number of hidden neurons as 12 through the trial from a small number up to the value when the decrease of the sum-squared network error became steady). In Figure 7, normalization function *f*_in_, denormalization function *f*_out_, sigmoid function of the hidden layer *f*_hid_ and transfer matrix *w*^ih^, *w*^ho^ constitute the model structure of the neural network function. The neural network model is developed through training using sample data. The training algorithm we used is the standard back propagation learning algorithm with the learning rate of 0.005 and the momentum parameter of 0.5 to find the network parameters that minimize the errors between the network outputs and actual values.

**Figure 8.**
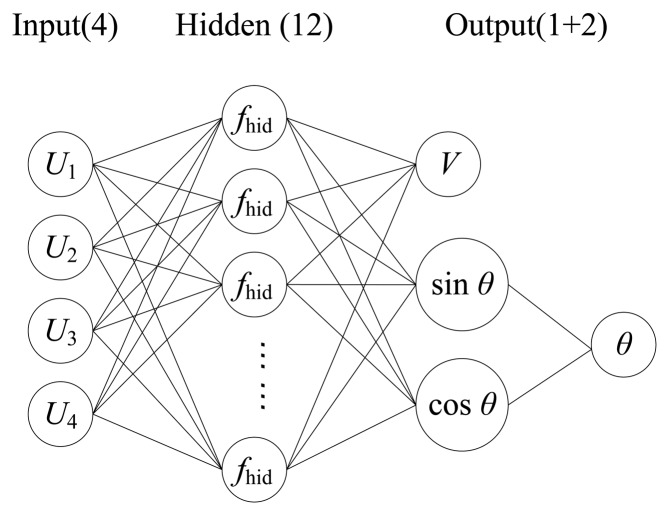
Improved network structure with sine and cosine as outputs.

When establishing the BP neural network, we found that the direction angle of the flow vector was not a good output variable for the network because when the vector angle varies from 0° to 360°, the output of the sensing element follows approximately sine or cosine law that is not consistent with the monotonicity of angle variation. Therefore, we considered to substitute sine and cosine of the direction angle for the angle itself as the outputs of the network to simplify the calculation as shown in Figure 8. The flow direction can be figured out by synthesizing sin*θ* and cos*θ*.

## 4. Wind Tunnel Experiments and Analysis

### 4.1. Experimental Setup

For validating the effectiveness of the flow sensor, we conducted two experiments including a calibration experiment and a measuring experiment using a low-turbulence wind tunnel in the airspeed range of 3–30 m/s with typical error of less than 1.5% full scale (FS). The sensor was mounted on a turntable installed in the wind tunnel. The flow speed is adjusted between 3–30 m/s and the turntable was adjusted from 0 to 360° with accuracy better than 0.1°. The experiments were conducted at room temperature around 20 °C. Reynolds number for the airflow range 3–30 m/s is from 14,000 to 150,000.

The sensor signals were collected using a PC via 12-bit AD device. Two independent experiments were conducted, one was for developing/training the neural network model (calibration) and the other one was for evaluating the sensor (measurement). In the calibration experiment, model development was conducted by adjusting the airflow speed to 3, 6, 9, … and 30 m/s (in 3 m/s per step), respectively. At each constant flow speed, the turntable with the sensor prototype was rotated from 0° to 360° at 10° per step while outputs of the four sensing elements were recorded. The parameters of the BP network were determined through training, where the output data of the sensor elements were used as inputs and the corresponding data of the flow speed and direction were used as the outputs of the network. After 10,000 epochs of network training, the sum-squared network error was decreased to less than 0.1.

The performance of the sensor was evaluated through the measurement experiment. The developed network model was used for calculating the flow parameters in this experiment. The experiment was conducted by adjusting the flow speed again to 3, 6, 9, … and 30 m/s respectively, and at each flow speed the turntable with the sensor prototype was rotated from 5° to 355° at 10° per step that were different from the allocation of the direction angles in the calibration experiment. At each given instant, the readouts of the four elements of the sensor were imported into the developed BP network model and the flow speed and direction angle were exported from the network model online, and the values were compared with the actual values.

### 4.2. Experimental Results and Discussion

**Figure 9.**
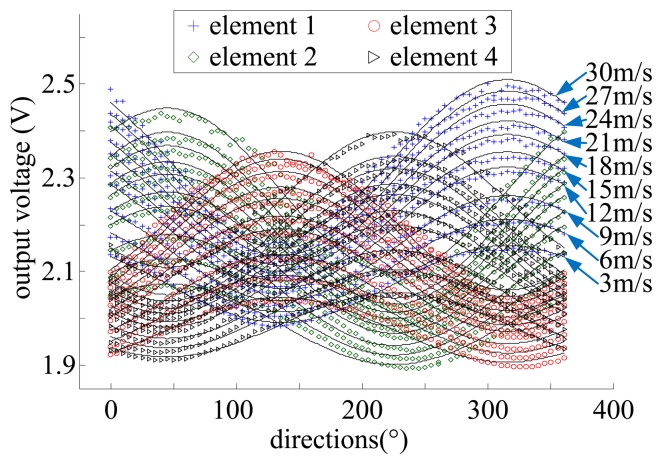
Output voltages of sensor versus the flow speed and flow direction (flow speeds are labeled for element 1 only).

**Figure 10.**
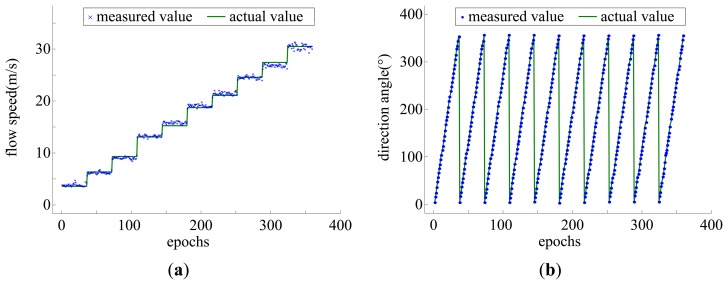
Measurement results, (**a**) for flow speeds and (**b**) direction angles.

Figure 9 shows the outputs of the sensing elements *versus* the flow speed of 3–30 m/s and the flow direction varying in the range of 0–360°. The data acquired in the calibration experiment were used for developing the model while the data acquired in the measurement experiment were used for evaluating the performance of the sensor by comparing the model-based outputs of the sensor with the actual flow parameters. The measurement results are shown in Figure 10.

From the results of Figure 10, it can be seen that the model-based measuring flow parameters fit the actual parameters very well. The mean square error of the airflow speed measurement reaches 0.72 m/s in the range of 3–30 m/s and the mean square error of the airflow direction measurement reaches 1.9° in the range of 0–360°.

## 5. Conclusions

A novel methodology for 2-D flow measurement using a simple round-shaped sensor and a neural network-based data fusion technology is proposed in this paper. The sensor is composed of four thermal roundabout wire elements, each of which serves as both Joule heater and temperature sensor. The configuration and the fabrication are simple and low cost. Wind tunnel experiments validated the effectiveness of the proposed sensor and showed that the measurement mean square errors reach 0.72 m/s for the airflow speed measurement in the range of 3–30 m/s and 1.9° for the airflow direction measurement in the range of 0–360°.
